# Sexuality and society in the medical context: Conceptualization, implementation and evaluation of a student-led elective course in medical school at Leipzig University

**DOI:** 10.3205/zma001763

**Published:** 2025-06-16

**Authors:** Konrad Jakob Endres, Chiara Surber, Mona Albertus, Martina Müller, Laura Wortmann, Nick Heinz, Ines Conrad, Heide Götze

**Affiliations:** 1 Leipzig University, Medical Faculty, Institute for Social Medicine, Occupational Medicine and Public Health (ISAP), Leipzig, Germany; 2 Leipzig University, Medical Faculty, Department of Medical Psychology and Medical Sociology, Leipzig, Germany; 3 Eating Disorders Counseling Center (BEL), Leipzig, Germany; 4 University of Bielefeld, Medical Faculty EWL, Department of Sex- and Gender-Sensitive Medicine, Bielefeld, Germany; 5 Trans-Inter-Active in Central Germany (TIAM), Leipzig, Germany

**Keywords:** sexual health, sexual and gender minorities, medical education, interactive learning, sexually transmitted infections, gender identity

## Abstract

**Objectives:**

Physicians often avoid discussing sexual and gender-related health with patients due to discomfort and fear of causing offense, leading to incomplete sexual histories and gaps in care, particularly affecting LGBTQI+ patients who face significant health disparities. This project aimed to address this gap by introducing an elective course titled “Sexuality and Society in the Medical Context” to provide German medical students with the skills and knowledge necessary for effective sexual health discussions.

**Methods:**

The course utilized interactive teaching methods, including peer learning and role-playing. Topics covered ranged from sexually transmitted infections to gender diversity and LGBTQI+ health. Student feedback was collected through a structured evaluation to assess the course’s impact and effectiveness.

**Results:**

Student feedback was highly positive; with participants highlighting the course’s engaging format and relevance to their medical education. The evaluation showed high satisfaction with the course's organization, content, and interactive elements.

**Conclusion:**

Although the elective course was well received, its limited scope suggests the need to integrate similar training into the core medical curriculum. This integration is crucial for enabling future physicians to systematically address sexual health issues, overcome systematic biases, and foster a supportive environment for all patients.

## Zusammenfassung

**Zielsetzung:**

Ärzt:innen vermeiden es häufig mit ihren Patient:innen über sexuelle und geschlechtsspezifische Fragen zu sprechen, weil es ihnen unangenehm ist oder sie Angst haben, Anstoß zu erregen. Dies führt häufig zu einer unvollständigen Erhebung der Sexualanamnese und zu Lücken in der Versorgung, insbesondere für LGBTQI+-Patient:innen, die mit erheblichen gesundheitlichen Benachteiligungen konfrontiert sind. Das beschriebene Projekt adressierte diese Lücke, indem ein Wahlfach mit dem Titel „Sexualität und Gesellschaft im Kontext der Medizin“ eingeführt wurde, um Medizinstudierenden die Fähigkeiten und das Wissen zu vermitteln, die für effektive Gespräche über sexuelle Gesundheit erforderlich sind.

**Methoden:**

Im Kurs wurden interaktive Lehransätze wie Peer-Learning und Rollenspiele eingesetzt. Thematisch wurden Bereiche wie sexuell übertragbare Infektionen, geschlechtliche Vielfalt und die Gesundheit von LGBTQI+-Personen abgedeckt. Es erfolgte eine strukturierte Evaluation des Wahlfaches.

**Ergebnisse:**

Das Feedback der Studierenden war äußerst positiv; die Teilnehmer*innen hoben besonders das ansprechende Format des Kurses und die Relevanz für ihre medizinische Ausbildung hervor. Die Evaluation zeigte eine hohe Zufriedenheit mit der Organisation, dem Inhalt und den interaktiven Elementen des Kurses.

**Schlussfolgerung:**

Trotz der positiven Resonanz ist die Reichweite eines Wahlfaches eingeschränkt. Ähnliche Inhalte sollten in den zentralen Lehrplan des Medizinstudiums aufgenommen werden. Dies ist unerlässlich, um angehenden Ärzt:innen zu helfen, Fragen der sexuellen Gesundheit systematisch anzugehen, Vorurteile zu überwinden und eine unterstützende Umgebung für alle Patient:innen zu schaffen.

## 1. Introduction

Physicians often avoid discussing sexual and gender-related health with patients due to discomfort and fear of offending, despite its importance for overall well-being [1]. This reluctance can lead to inadequate sexual histories and gaps in patient care [2]. Nevertheless, clinicians frequently encounter situations in their practice where addressing sensitive topics like sexual and gender-related health is essential, yet these conversations are not a routine part of patient care [3]. Furthermore, many health care professionals feel ill-prepared for these conversations and are typically reluctant or unskilled in sexual problem management [4]. However, it is important to keep in mind that most patients welcome physicians exploring sexual and gender-related health during consultations if an appropriate framework of trust is in place [5].

Although many medical professionals often feel uncertain and unprepared when addressing sexual and gender-related health, this uncertainty is especially pronounced in interactions with queer patients [6]. A survey from 2021 estimates that around 11% of the German population identifies as lesbian, gay, bisexual, trans*, queer, or intersex (LGBTQI+), underlining the relevance of health care of sexual minorities to public health [7]. Awareness of gender diversity, transgender individuals, queer people, and sexual minorities has grown significantly in recent years, both in the public sphere and within the media and among government representatives. The number of scientific studies has also risen sharply in the last 10 years (example: the number of publications in PubMed for the search terms “queer health” was N=182 in 2013, in 2023 it was N=3,182/for the search terms “sexual minorities” was N=308 in 2013, in 2023 it was N=3,975). Although gender diversity and sexual diversity have been increasingly depathologized in recent years, LGBTQI+ people are still confronted with discrimination, prejudice and, in some cases, inappropriate care, including in the German healthcare system [8], [9].

Sexual minorities often experience poorer physical and mental health compared to the general population [10]. For instance, sexual minority men and women exhibit a higher prevalence of conditions such as asthma, hypertension, and other diseases compared to their heterosexual counterparts [11], [12]. Possible reasons for these worse health outcomes are social rejection, lack of family acceptance, and discrimination at work [13]. A meta-analysis revealed that sexual minority youth are nearly three times more likely to experience suicidality [14]. Additionally, transgender adolescents often report having eating disorders, in some cases as an attempt to stop the development of secondary sexual characteristics during puberty or to change their body shape [15], [16]. Sexual minorities also report worse healthcare experiences, including less trust in doctors and lower satisfaction with care [17]. While social factors clearly contribute to mental health disparities, the relationship with physical conditions such as asthma is more complex. Although the exact mechanisms are not fully understood, research suggests that psychosocial factors, particularly increased stress related to sexual minority status, may contribute to the higher prevalence of asthma in the population of sexual minorities [18].

This underscores the need for educational opportunities in medical school that focus on health of the queer community and sexual minorities. To achieve this, it is particularly important to address the social determinants of health and illness, especially in the context of sexual health. Future physician training should therefore concentrate on demonstrating the relevance of sexual health across medical disciplines and helping physicians overcome their discomfort in discussing these topics. Unfortunately, courses at medical faculties that provide practical and interactive training on topics such as sexual health and conducting a sexual history are still scarce [19], [20]. As a result, students often graduate without the necessary skills to address these issues confidently and professionally in their future careers. Research shows that medical students feel inadequately trained to address sexual health concerns despite recognizing its importance [21]. Closing this knowledge gap could be achieved by developing structured educational programs for medical students that cover these topics.

We hypothesize that an elective course on gender diversity and sexual health in medical education will result in high satisfaction rates among both preclinical and clinical students, and receive positive evaluations from participants.

## 2. Project description and methods

### 2.1. Development of the project

The course development originated from the lecture series “Medizin Divers”, and focused on the concerns of vulnerable patient groups and was initiated by students from the university group “Mit Sicherheit Verliebt” [https://www.bvmd.de/projekte-und-ags/projekte/mit-sicherheit-verliebt/das-projekt/]. Following the lecture series, the idea emerged to solidify these topics further within the university curriculum. Additionally, tutors have been trained in LSTBQ*-inclusive sexual history taking and role-plays focusing on STIs by Deutsche Aidshilfe via the training program “Let’s talk about Sex”. “Mit Sicherheit Verliebt” is an initiative of the Federal Representation of Medical Students in Germany (Bundesvertretung der Medizinstudierenden in Deutschland e.V.) and conducts sexual education workshops in German schools. Other local groups of “Mit Sicherheit Verliebt” have also established elective courses at their respective faculties, such as in the medical faculties of Rostock and Munich.

### 2.2. Learning objectives

The course aims to give students the knowledge and skills they need to handle the complex issues surrounding sexual and gender-related health in medical settings. The course has several important learning objectives that are based on the National Competency-Based Learning Objectives Catalogue for Medicine, Version 2.0. 

Firstly, students learn about sexually transmitted infections (STIs). They can describe them, including how to prevent them, how to diagnose them accurately and how to treat them effectively (ID in NKLM 2.0: VII.3-19.2.15). After the elective course, students are able to take and document a sexual anamnesis (VIII.2-02.4.7). The students also learn to be respectful and inclusive when caring for patients with different sexual orientations and identities. They can identify and articulate sensitive sexual topics in everyday medical practice, conduct appropriate discussions or consultations sensitively and in accordance with current standards and structure their communication appropriately even in emotionally challenging situations (VIII.2-3). Participating students can perceive taboo sexual topics and stigmatization and, if it seems sensible or necessary to delve deeper, address this topic appropriately (VIII.2-03.2.6). They reflect on subjective processes of sexual health and illness in their interactions with individual and social conditions (NKLM 2.0 VII.1a-02.6.6). In essence, students develop a basic medical attitude of compassion in the course and learn to provide attentive care for their patients. 

However, the primary objective of this article is to assess the subjective satisfaction of the students with the course. We did not focus on evaluating the achievement of specific learning objectives.

### 2.3. Structure and organization

The course comprised eleven two-hour sessions and was designed for two separate cohorts of 10 preclinical students and 10 clinical students. The preclinical part was conducted through the Department of Medical Psychology and Medical Sociology, while the Institute for Social Medicine, Occupational Medicine and Public Health (ISAP) at Leipzig University managed the clinical part.

For the organization and implementation of the elective course, the Institute for Social Medicine, Occupational Medicine and Public Health (ISAP) provided two positions for student assistants. The Department of Medical Psychology and Medical Sociology of Leipzig University provided a further student assistant position and a budget for external lecturers.

The selection process for this elective course followed a first-come, first-served approach. Students were chosen based on the order in which they applied. Students in the preclinical phase had to register via email and students in the clinical phase via a registration link.

### 2.4. Didactic concept

#### 2.4.1. Interactive format, peer-teaching and role-plays

The elective course used a teaching method called peer teaching, where students learned from each other and the instructor. This was achieved through numerous discussions and group activities where everyone could participate. Instead of the lecturer dominating the session with a traditional lecture format, time was ensured for students to share their ideas and ask questions during and after each lesson. We selected the peer-assisted learning (PAL) model for several key reasons. PAL creates a more relaxed, open learning environment, and allows more interactive and participatory learning. Research supports these benefits, showing PAL helps with the social aspects of learning, and is valued for consolidating knowledge and improving professional skills including collaboration and feedback [22], [23].

For the session on sexual history, three role-playing exercises were conducted to practically address aspects of the doctor-patient relationship and communication. In these exercises, three different scenarios were played out in small groups, each supervised by a tutor. Each student had the opportunity to assume three different roles: once as the physician, once as the patient, and once as an observer providing feedback. The roles were rotated so that all students actively participated in the role-play at least once, either as a physician or as a patient, ensuring a comprehensive learning experience from multiple perspectives and enhancing their understanding of the doctor-patient dynamic in various sexual health contexts. With friendly permission from the Deutsche Aidshilfe, the role-playing templates from the “Let’s Talk About Sex” training program were used [https://www.hiv-sti-fortbildung.de/en]. 

#### 2.4.2. Sessions and covered topics

Table 1 (Tab. 1) includes the educational methods and covered topics for the eleven two-hour sessions of the elective course. Each session included different activating exercises at the start and throughout the session. 

#### 2.4.3. An interdisciplinary and interprofessional approach

In the elective course, an interdisciplinary and interprofessional approach was emphasized. Collaboration was established with experts from diverse domains, including Martina Müller, an educational scientist from the Eating Disorders Counseling Center in Leipzig (BEL), Dr. Nick Heinz, an expert on trans, inter and nonbinary health from Trans-Inter-Active in Central Germany (TIAM) in Saxony, and Dr. Laura Wortmann, a physician and research associate in the Department of Sex- and Gender-Sensitive Medicine at the Faculty of Medicine, University of Bielefeld.

#### 2.4.4. Proof of performance 

Students were required to give a presentation on a self-chosen topic. The evaluation was conducted by lecturers from the University of Leipzig in the fields of social medicine and medical psychology, in consultation with student assistants. Presentations could be either individual or group presentations, with a maximum of three people per group. The examination presentations covered the following topics: queer and gender-sensitive questionnaires in doctors’ offices, LGBTQ-friendly practices, medication development and its test subject demographics, erectile dysfunction and female sexual dysfunction (FSD), transgender pregnancies, female genital mutilation, fertility desires of non-heterosexual/non-cisgender couples and research on hormonal contraception methods for different genders.

### 2.5. Evaluation 

The evaluation of the elective course was conducted using a structured questionnaire with items grouped into three categories: organization, structure, and personal benefit. Respondents were asked to rate each item on a six-point Likert scale, with response options ranging from “strongly agree” to “strongly disagree”. In addition to the structured items, the questionnaire included two open-ended questions: “What did you particularly like about the course?” and “What did you not like, and what should be improved?” These open-ended questions provided qualitative data on student experiences and suggestions for course improvement. Furthermore, the students were asked to rate the course overall, using a rating scale from 1 to 6. A rating of “1” corresponded to “very good” and a rating of “6” corresponded to “unsatisfactory”, following the German school grading system.

The questionnaire was developed by the Teaching Department of the Faculty of Medicine at Leipzig University and was systematically evaluated there. The questionnaire was distributed to the students during the final session of the elective course. The evaluation was conducted anonymously and voluntarily and the questionnaire can be found in the attachment 1 .

## 3. Results of the evaluation

A total of 17 students participated in the evaluation, however not all items received responses from each participant, leading to differing sample sizes for specific questions (see table 2 (Tab. 2)).

The evaluation of the elective course showed that the students rated all of the items very well (see table 2 (Tab. 2)). Notably, the interactivity of the course was rated with a mean score of 1.0 (SD=0.0), the perceived importance for their medical education received a mean score of 1.1 (SD=0.2) and the overall course rating was also excellent, with a mean score of 1.0 (SD=0.0) (see table 2 (Tab. 2)). These high scores reflect strong student satisfaction. Additionally, it is noteworthy that there were more than five applicants for each available spot in this elective course during the preclinical phase, underscoring its popularity and demand among students.

In the free-text responses of the evaluation questionnaire, students were also asked for suggestions for improvement. Analysis of the free-text responses revealed that some students would have preferred the course to be scheduled on weekdays, expressed interest in including endometriosis as a topic, and wanted deeper exploration of some subjects. In response to the open questions about what was particularly good, the following aspects were frequently mentioned: interactive and diverse teaching methods, variety and engagement in the coursework, peer-to-peer approach, open discussions and positive classroom atmosphere, non-discriminatory language, practical relevance, space for topics typically covered briefly in the curriculum, and dedicated tutors or student assistants. The evaluation was conducted and analyzed by the Office of Teaching, Medical Faculty, University of Leipzig.

## 4. Discussion

In sum, the elective course “Sexuality and Society in the Medical Context” was rated very well in the domains of organization, structure and personal benefit and achieved a very good overall rating. In the free-text-responses, the main concepts of the elective course (interactivity and peer-to-peer approach), were highlighted positively. From these results, we conclude that student satisfaction was high, aligning with our initial hypothesis. 

The excellent evaluation, learning benefit reported by the students and the fact that there were more than five applicants for each available spot in the preclinical phase of medical school demonstrate the high demand for further educational opportunities that cover the topic of sex and gender diversity and sexual health and use interactive methods to convey these topics. This finding is further corroborated by a nation-wide survey of over 3264 medical students from all 37 medical schools in Germany, where a majority of the respondents answered that courses in sexual health should be mandatory in medical school [24].

Besides the focus on sexuality in the medical context, a session for gender medicine and gender education was conducted in the elective course (see table 1 (Tab. 1)). Research revealed that medical students consider sex/gender and culture-sensitive competencies relevant to their education [25]. Furthermore, a quantitative cross-sectional survey at four German medical schools (N=750) has shown that the implementation of gender medicine courses in the medical curriculum has a significant impact in students’ gender competence [26]. Thus, for future courses at medical school, integrating sexual medicine with gender medicine topics could be particularly interesting, because the two topics overlap significantly.

This teaching evaluation has a number of limitations. First, an important limitation of the results is that only a limited number of students (N=20) could participate in the course due to its elective format. The results of the evaluation (N=17) can only be generalized to the overall cohort of medical students to a very limited extent, as the sample size is relatively small and usually students that are already interested in the topics take part in the electives. This introduced bias and could lead to a false positive assessment of the elective. Integrating sexual health courses into the core curriculum, making them mandatory, and incorporating continuous student evaluations could address this issue. Implementation into the compulsory curriculum at medical schools could also show the long-term effects of sexual education in medical school. Furthermore, the questionnaire focused on students’ evaluation of the course rather than directly assessing whether the learning objectives were achieved, limiting our ability to measure the actual impact of the teaching intervention. For future medical school sexual education programs, it is important to assess medical students' attitudes and knowledge of sexuality-related topics before and after the courses in order to evaluate the potential impact of the teaching opportunity. Lastly, students tended to evaluate each other very positively during the role-playing exercises, which may be attributed to a collegial bias among peers. It could be beneficial to employ actors as simulated patients, providing students with more realistic and potentially challenging scenarios to navigate. The role-playing sessions could be video recorded, allowing for more detailed and objective feedback. This would enable students to review their performances, making the feedback process more comprehensive and easier to understand.

A student-led elective course has several advantages and disadvantages. Advantages include the introduction of fresh perspectives and contemporary topics, peer engagement, fostering an inclusive learning environment and encouraging students to participate actively and take ownership of their education. Disadvantages include sustainability issues as the course’s continuity can be jeopardized if student assistants graduate, and limited funding resources. Similarly to other student-led elective courses, the topics of elective courses can only have a long-term perspective in medical education if they are included in the main curriculum as a compulsory component in the catalog of learning objectives and medical curricula, institutionalized, and designed and implemented by university staff [27]. In particular, the topics of the elective course “Sexuality and Society in the Medical Context” can be a valuable addition to the already existing catalog of learning objectives of medical psychology, or, for example, to communication curricula.

From the perspective of the student tutors, the experience of the elective course was highly enriching and engaging, with students showing significant interest and contributing actively throughout the course. The positive learning atmosphere and enthusiastic participation of the students were particularly gratifying. To further enhance the learning experience in future iterations, the student tutors would consider providing more online materials.

## 5. Conclusion

In summary, the elective course “Sexuality and Society in the Medical Context” was highly sought after and positively evaluated due to its important topics, which are often inadequately addressed in traditional medical education curricula, and its interactive teaching methods. The positive evaluation demonstrated a demand for such educational opportunities, which not only cover aspects of sexual health and social determinants of health and disease but also employ interactive teaching methods such as peer teaching and practical exercises. As an implication for medical education, similar courses should be integrated not only as electives but also into the mandatory curriculum. This integration is essential so that future physicians can explore these topics during their studies, enabling them to confidently take a systematic sexual history later in their careers, overcome implicit biases, and create a welcoming, safe environment for all those seeking help [28]. 

## Notes

### Funding

Supported by the Open Access Publishing Fund of Leipzig University.

### Authors’ ORCIDs

- Konrad Jakob Endres: [0009-0008-7306-8782]
- Laura Wortmann: [0000-0002-3210-0474]
- Ines Conrad: [0000-0002-9829-2210]
- Heide Götze: [0000-0003-3056-5822]

## Competing interests

The authors declare that they have no competing interests. 

## Supplementary Material

Questionnaire (only in German)

## Figures and Tables

**Table 1:**
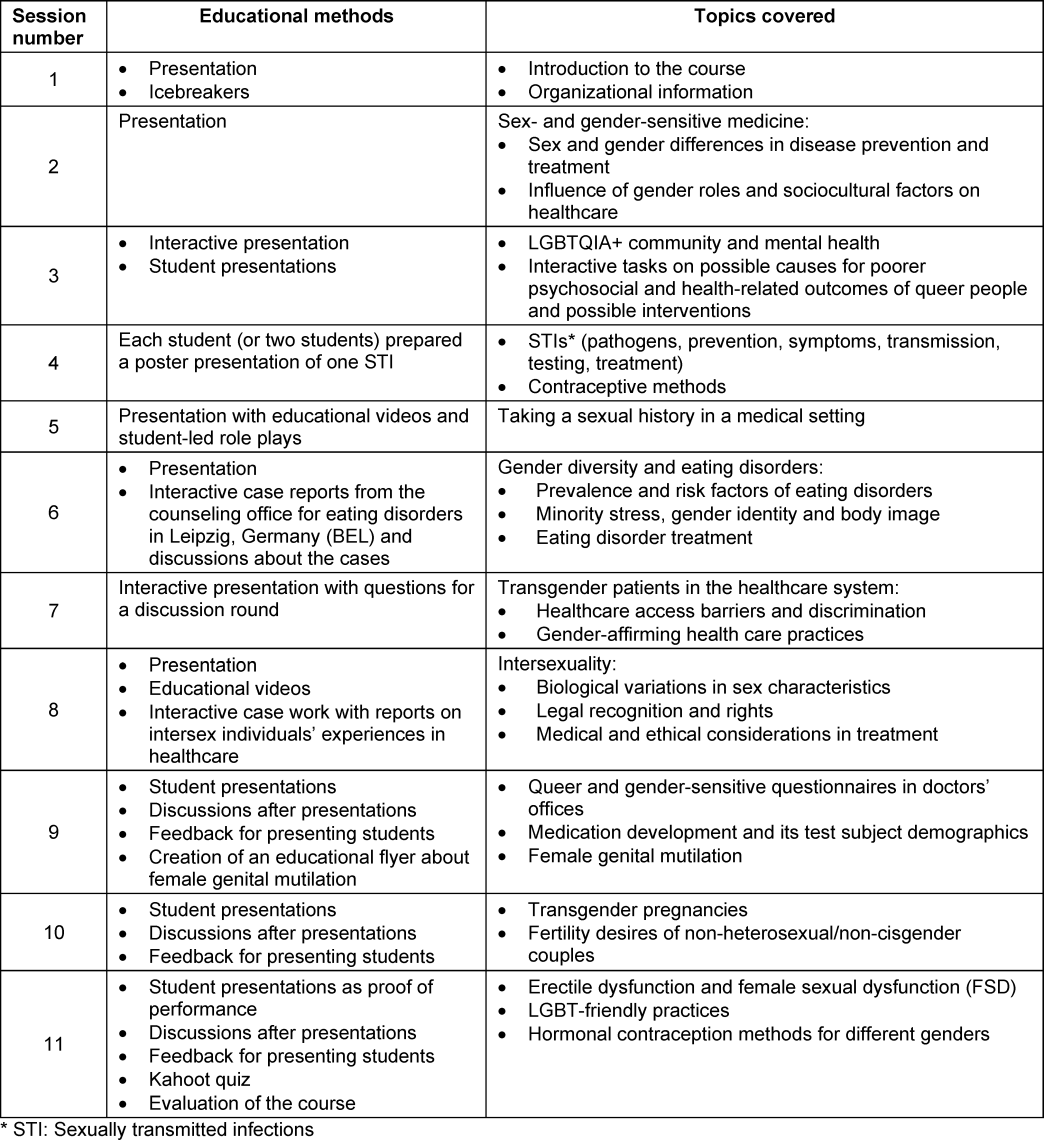
Summary of sessions with educational methods and covered topics

**Table 2:**
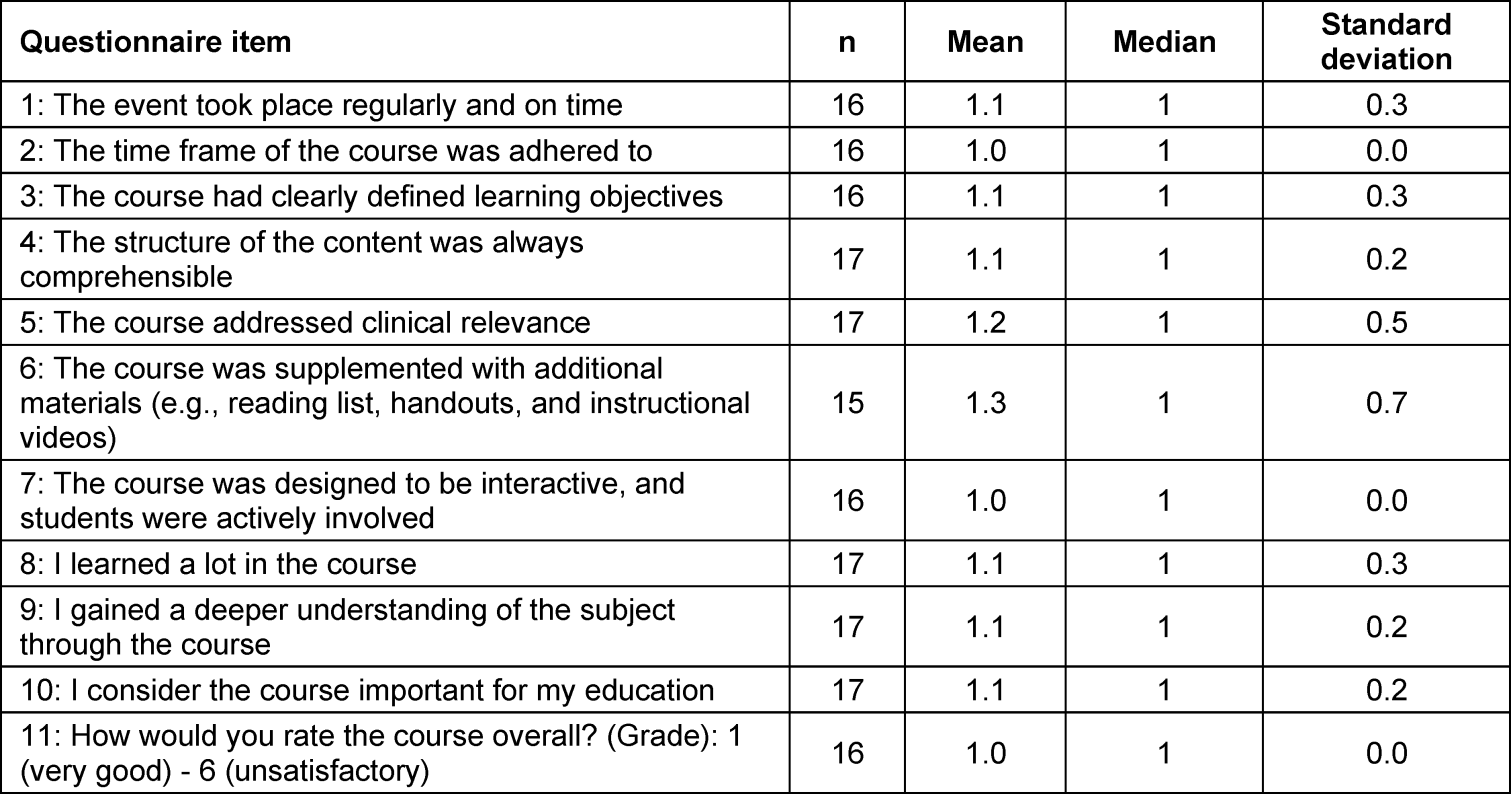
Evaluation of the elective course by the participating students (scale ranges from 1=strongly agree to 6=strongly disagree)

## References

[1] Komlenac N, Hochleitner M (2020). Predictors for Low Frequencies of Patient-Physician Conversations Concerning Sexual Health at an Austrian University Hospital. Sex Med.

[2] Brookmeyer KA, Coor A, Kachur RE, Beltran O, Reno HE, Dittus PJ (2021). Sexual History Taking in Clinical Settings: A Narrative Review. Sex Transm Dis.

[3] Meystre-Agustoni G, Jeannin A, de Heller K, Pécoud A, Bodenmann P, Dubois-Arber F (2011). Talking about sexuality with the physician: are patients receiving what they wish?. Swiss Med Wkly.

[4] Althof SE, Rosen RC, Perelman MA, Rubio-Aurioles E (2013). Standard Operating Procedures for Taking a Sexual History. J Sex Med.

[5] Zéler A, Troadec C (2020). Doctors Talking About Sexuality: What Are the Patients’ Feelings?. Sex Med.

[6] Dyer K, das Nair R (2013). Why Don’t Healthcare Professionals Talk About Sex? A Systematic Review of Recent Qualitative Studies Conducted in the United Kingdom. J Sex Med.

[7] Ipsos (2021). LGBT+ Pride 2021 Global Survey.

[8] Wittlin NM, Kuper LE, Olson KR (2023). Mental Health of Transgender and Gender Diverse Youth. Annu Rev Clin Psychol.

[9] Pang KC, de Graaf NM, Chew D, Hoq M, Keith DR, Carmichael P, Steensma TD (2020). Association of Media Coverage of Transgender and Gender Diverse Issues With Rates of Referral of Transgender Children and Adolescents to Specialist Gender Clinics in the UK and Australia. JAMA Netw Open.

[10] Mereish EH, Poteat VP (2015). A relational model of sexual minority mental and physical health: The negative effects of shame on relationships, loneliness, and health. J Couns Psychol.

[11] Haarmann L, Folkerts AK, Lieker E, Eichert K, Meidlinger M, Monsef I, Skoetz N, Träuble B, Kalbe E (2023). Comprehensive systematic review and meta-analysis on physical health conditions in lesbian- and bisexual-identified women compared with heterosexual-identified women. Womens Health.

[12] Haarmann L, Lieker E, Folkerts AK, Eichert K, Neidlinger M, Monsef I, Skoetz N, Träuble B, Kalbe E (2024). Higher Risk of Many Physical Health Conditions in Sexual Minority Men: Comprehensive Systematic Review and Meta-Analysis in Gay- and Bisexual-Identified Compared with Heterosexual-Identified Men. LGBT Health.

[13] Drydakis N (2022). Social Rejection, Family Acceptance, Economic Recession, and Physical and Mental Health of Sexual Minorities. Sex Res Soc Policy.

[14] Marshal MP, Dietz LJ, Friedman MS, Stall R, Smith HA, McGinley J, Thoma BC, Murray PJ, D’Augelli AR, Brent DA (2011). Suicidality and Depression Disparities Between Sexual Minority and Heterosexual Youth: A Meta-Analytic Review. J Adolesc Health.

[15] Coelho JS, Suen J, Clark BA, Marshall SK, Geller J, Lam PY (2019). Eating Disorder Diagnoses and Symptom Presentation in Transgender Youth: a Scoping Review. Curr Psychiatry Rep.

[16] Romito M, Salk RH, Roberts SR, Thoma BC, Levine MD, Choukas-Bradley S (2021). Exploring transgender adolescents’ body image concerns and disordered eating: Semi-structured interviews with nine gender minority youth. Body Image.

[17] Elliott MN, Kanouse DE, Burkhart Q, Abel GA, Lyratzopoulos G, Beckett MK, Schuster MA, Roland M (2015). Sexual Minorities in England Have Poorer Health and Worse Health Care Experiences: A National Survey. J Gen Intern Med.

[18] Heck JE, Jacobson JS (2006). Asthma diagnosis among individuals in same-sex relationships. J Asthma.

[19] Kemble J, Köhler T, Helo S, Warner JN, Ziegelmann M (2023). (216) Insufficient Medical School Sexual Health Curriculum Leads to Inadequately Prepared Trainees. J Sex Med.

[20] Brandt G, Stobrawe J, Korte S, Prüll L, Laskowski NM, Halbeisen G, Paslakis G (2022). Medical Students’ Perspectives on LGBTQI+ Healthcare and Education in Germany: Results of a Nationwide Online Survey. Int J Environ Res Public Health.

[21] Wittenberg A, Gerber J (2009). Recommendations for Improving Sexual Health Curricula in Medical Schools: Results from a Two-Arm Study Collecting Data from Patients and Medical Students. J Sex Med.

[22] Hammond JA, Bithell CP, Jones L, Bidgood P (2010). A first year experience of student-directed peer-assisted learning. Act Learn High Educ.

[23] Sevenhuysen S, Farlie MK, Keating JL, Haines TP, Molloy E (2015). Physiotherapy students and clinical educators perceive several ways in which incorporating peer-assisted learning could improve clinical placements: a qualitative study. J Physiother.

[24] Turner D, Nieder TO, Dekker A, Martyniuk U, Herrmann L, Briken P (2016). Are medical students interested in sexual health education? A nationwide survey. Int J Impot Res.

[25] Ludwig S, Dettmer S, Wurl W, Seeland U, Maaz A, Peters H (2020). Evaluation of curricular relevance and actual integration of sex/gender and cultural competencies by final year medical students: effects of student diversity subgroups and curriculum. GMS J Med Educ.

[26] Wortmann L, Haarmann L, Yeboah A, Kalbe E (2023). Gender medicine teaching increases medical students’ gender awareness: results of a quantitative survey. GMS J Med Educ.

[27] Fülbert H, Schäfer LN, Gerspacher LM, Bösner S, Schut C, Krolewski R, Knipper M (2023). Elective course “Climate-sensitive health counselling” – prevention as an opportunity for people and planet? An interactive, student-led project focusing on prevention and agency in physician’s climate communication. GMS J Med Educ.

[28] Waryold JM, Kornahrens A (2020). Decreasing Barriers to Sexual Health in the Lesbian, Gay, Bisexual, Transgender, and Queer Community. Nurs Clin North Am.

