# Supplementary material for: Sexuality and society in the medical context: Conceptualization, implementation and evaluation of a student-led elective course in medical school at Leipzig University
Source: GMS J Med Educ. 2025 Jun 16;42(3):Doc39. doi: 10.3205/zma001763 (PMC12286872; doi:10.3205/zma001763)
Supplement: Questionnaire (only in German) [file JME-42-39-s-001.pdf]

Attachment 1: Questionnaire (only in German)

Attachment 1 to Endres KJ, Surber C, Albertus M, Müller M, Wortmann L, Heinz N, Conrad I, Götze H. *Sexuality and society in the medical context: Conceptualization, implementation and evaluation of a student-led elective course in medical school at Leipzig University.* GMS J Med Educ. 2025;42(3):Doc39. DOI: 10.3205/zma001763

Medizinische Fakultät der Universität Leipzig

SS 2024 Wahlfach "Sexualität und Gesellschaft"

Bitte so markieren: ☐ ☒ ☐ ☐ ☐ Bitte verwenden Sie einen Kugelschreiber oder nicht zu starken Filzstift. Dieser Fragebogen wird maschinell erfasst.

Korrektur: ☐ ■ ☐ ☒ ☐ Bitte beachten Sie im Interesse einer optimalen Datenerfassung die links gegebenen Hinweise beim Ausfüllen.

Inwieweit stimmen Sie den folgenden Aussagen über die Lehrveranstaltung zu?

1. Allgemeines / Organisation

		stimme voll und ganz zu					stimme überhaupt nicht zu
1.1	Die Veranstaltung fand regelmäßig und pünktlich statt.	☐	☐	☐	☐	☐	☐
1.2	Der zeitliche Rahmen der Lehrveranstaltung wurde eingehalten.	☐	☐	☐	☐	☐	☐

2. Aufbau und Struktur

		stimme voll und ganz zu					stimme überhaupt nicht zu
2.1	Die Lehrveranstaltung hatte klar definierte Lernziele.	☐	☐	☐	☐	☐	☐
2.2	Die Gliederung des Stoffes (roter Faden) war stets nachvollziehbar.	☐	☐	☐	☐	☐	☐
2.3	In der Veranstaltung wurde auf den klinischen Bezug eingegangen.	☐	☐	☐	☐	☐	☐
2.4	Die Veranstaltung wurde durch weitere Materialien (z.B. Literaturliste, Handout, Lehrvideos) ergänzt.	☐	☐	☐	☐	☐	☐
2.5	Die Lehrveranstaltung wurde interaktiv gestaltet und die Student:innen aktiv einbezogen.	☐	☐	☐	☐	☐	☐

3. Persönlicher Nutzen

		stimme voll und ganz zu					stimme überhaupt nicht zu
3.1	Ich lernte viel in der Veranstaltung.	☐	☐	☐	☐	☐	☐
3.2	Ich gewann durch die Veranstaltung ein tieferes Verständnis des Stoffs.	☐	☐	☐	☐	☐	☐
3.3	Ich schätze die Veranstaltung als wichtig für meine Ausbildung ein.	☐	☐	☐	☐	☐	☐

4. Abschließende Bewertung

		1 sehr gut	2	3	4	5	6 ungenügend
4.1	**Wie würden Sie die Lehrveranstaltung abschließend bewerten? (Note):**	☐	☐	☐	☐	☐	☐

Bitte wenden!

5. **Lob / Verbesserungsvorschläge**

5.1 Was war besonders gut an dieser Lehrveranstaltung?

5.2 Was hat Ihnen nicht gefallen, was sollte verbessert werden?
